# *Leishmania (Viannia) guyanensis* Infection, Austria

**DOI:** 10.3201/eid1809.111365

**Published:** 2012-09

**Authors:** Wolfgang Poeppl, Heinz Burgmann, Herbert Auer, Gerhard Mooseder, Julia Walochnik

**Affiliations:** Military Hospital Vienna, Vienna, Austria (W. Poeppl, G. Mooseder);; and Medical University of Vienna, Vienna (W. Poeppl, H. Burgmann, H. Auer, J. Walochnik)

**Keywords:** Leishmania guyanensis, *Leishmania*, *Viannia*, *Phlebotomus*, *Lutzomyia*, Sandfly, leishmaniasis, parasites, Austria

**To the Editor:** Infection with *Leishmania* spp. was diagnosed in an asymptomatic soldier during an explorative national cross-sectional serologic screening of soldiers volunteering for United Nations missions at the Military Hospital Vienna in 2009. Diagnosis was made by using a commercial ELISA kit (Ridascreen *Leishmania*; R-Biopharm, Darmstadt, Germany). One year later, the soldier was reassessed for persisting antibodies by using the same ELISA and for *Leishmania* DNA in a blood sample stored in EDTA by using the *Leishmania* OligoC-Test (Coris BioConcept, Gembloux, Belgium). (The study was approved by the Research Ethics Committee of the Austrian Armed Forces and written informed consent was obtained from the person investigated.) Because the results of both tests were positive, an additional PCR was performed for identification below genus level with the LITSR/L5.8S primer pair (*1*). To confirm the PCR results, we sequenced the amplicon in both directions in 2 independent setups and compared the obtained 299-bp sequence with published sequences from GenBank by performing a multiple sequence alignment. Our sequence (strain EN10) showed 100% (299/299 bp) identity with several strains from the *Leishmania (Viannia) guyanensis* complex, including the *L. guyanensis* strain MHOM/SR/87/TRUUS4 and the *L. panamensis* strains FJ948438, FJ948439, and FJ948446. Sequence homology to representatives of the *L. (V.) braziliensis* complex was ≈93%; to representatives of the *L. (Leishmania) mexicana* complex, 61%–68%; and to the *L. (L.) donovani* complex, 70%–71%. The *L. (V.) guyanensis* complex traditionally includes the species *L. guyanensis*, *L. panamensis,* and *L. shawi*, but *L. panamensis* seems to be a subspecies or even a synonym of *L. guyanensis* (*2*). We thus classified our strain as *L. guyanensis*. Sequence data were deposited at GenBank (accession no. JN671917).

*L. guyanesis/panamensis* is found in 9 countries in Central and South America (*3*). It is a common cause of zoonotic cutaneous leishmaniasis in humans. The sloths *Choloepus didactylus* (*L. guyanensis*) and *C. hoffmanni* (*L. panamensis*) are believed to be the principal reservoir hosts and the sandfly species *Lutzomyia umbratilis* (*L. guyanensis*) and *Lu. trapidoi* (*L. panamensis*) to be the principal vectors (*3*). Also, dogs can act as reservoirs for the *L. (V.) guyanensis* complex (*4*).

The infected soldier had never been to Central or South America and had no history of blood transfusions. His lifetime travel history included Italy, Spain, Greece, Germany, Croatia, New York City, and military assignments in Kosovo. Thus, how and where the infection had been acquired remain open for discussion.

Although sandflies are not as robust as *Anopheles* spp., for example, the most plausible scenario is that either an *L. guyanensis*–infected sandfly or a noninfected but transmissive sandfly from a disease-endemic area was transported in a ship or airplane (comparable to the well-known “airport malaria” situation) to an area where the patient had traveled. In recent years, *Lu. vexator* has become widespread and abundant in upstate New York (*5*). Although, this is not a known vector for *L. guyanensis*, its spread in New York State shows that *Lutzomyia* spp. can rapidly adapt to new and distant areas. Of the areas where the infected person had traveled, at least in New York City and Spain, regular introduction of *L. guyanensis* by immigrants, travelers, or dogs from Central and South America is very likely. Moreover, *Leishmania* parasites are known to remain viable for a lengthy period in infected humans and animals and even in those that have received treatment.

However, alternative scenarios with other sandfly species, possibly even those found in Europe, acting as vectors cannot be totally excluded. Approximately 25 sandfly species are found in Europe, of which at least 6 are vectors for *Leishmania* spp (*6*). Whether *L. guyanensis* can be transmitted by *Phlebotomus* sandfly species is unknown. When *L. infantum*, originally transmitted by *Phlebotomus* spp., was introduced from Europe to Central and South America in the post-Columbian era, it readily adapted to several vectors of the genus *Lutzomyia* (*3*). Adoption of new reservoir hosts and new vector species has also been observed in other species (*7*). Members of the *Leishmania* subgenus develop in the midgut, and representatives of the *Viannia* subgenus develop in the hindgut and the midgut. Nevertheless, several *Lutzomyia* species can transmit both, representatives of the *Viannia* and *Leishmania* subgenera. In general, most sandflies appear to be vector competent for >1 *Leishmania* spp. The New World species *Lu. longipalpis* and the Old World species *Ph. argentipes*, *Ph. arabicus*, *Ph. halepensis,* and *Ph. perniciosus* enabled the maturation of almost all *Leishmania* species tested under experimental conditions (*8*). The presence of sandflies in Austria was reported very recently (*9*), but the vector competence of the species found (*P. mascittii*) has still not been elucidated. Moreover, this finding likely reflects an increased population density rather than an introduction of a previously nonendemic species.

Nonvector transmission is also a possibility. The infected person did not remember ever having received blood products; however, transmission is generally possible by all forms of blood contact, including through needle sharing among persons who use injection drugs and through sexual intercourse (*10*).
